# The Many Faces of Fear: Comparing the Pathways and Impacts of Nonconsumptive Predator Effects on Prey Populations

**DOI:** 10.1371/journal.pone.0002465

**Published:** 2008-06-18

**Authors:** Evan L. Preisser, Daniel I. Bolnick

**Affiliations:** 1 Department of Biological Sciences, University of Rhode Island, Kingston, Rhode Island, United States of America; 2 Section of Integrative Biology, University of Texas at Austin, Austin, Texas, United States of America; University of Zurich, Switzerland

## Abstract

**Background:**

Most ecological models assume that predator and prey populations interact solely through consumption: predators reduce prey densities by killing and consuming individual prey. However, predators can also reduce prey densities by forcing prey to adopt costly defensive strategies.

**Methodology/Principal Findings:**

We build on a simple Lotka-Volterra predator-prey model to provide a heuristic tool for distinguishing between the demographic effects of consumption (consumptive effects) and of anti-predator defenses (nonconsumptive effects), and for distinguishing among the multiple mechanisms by which anti-predator defenses might reduce prey population growth rates. We illustrate these alternative pathways for nonconsumptive effects with selected empirical examples, and use a meta-analysis of published literature to estimate the mean effect size of each pathway. Overall, predation risk tends to have a much larger impact on prey foraging behavior than measures of growth, survivorship, or fecundity.

**Conclusions/Significance:**

While our model provides a concise framework for understanding the many potential NCE pathways and their relationships to each other, our results confirm empirical research showing that prey are able to partially compensate for changes in energy income, mitigating the fitness effects of defensive changes in time budgets. Distinguishing the many facets of nonconsumptive effects raises some novel questions, and will help guide both empirical and theoretical studies of how predation risk affects prey dynamics.

## Introduction

Predators capture, kill, and consume their prey. This apparently trivial statement has complex and important implications: predators reduce prey population density, which in turn can affect the population growth of prey resources and other predators. The ‘cascading’ effects of consumption propagate throughout ecological communities, and are critically important to community dynamics. Food web models attempt to capture these dynamics using simultaneous population dynamic equations that link predators, prey, and resources via consumption rates [1]. Basic predator-prey models illustrate the primacy of consumption in structuring ecological thinking about food webs. For example, the classic Lotka-Volterra equations [2], [3] describe the interaction between predator (P) and prey (N) population densities as follows:
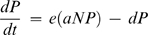
(1)

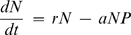
(2)where *a* is the capture rate, *e* is the rate at which offspring are produced per unit of energy income (*aNP*) into progeny, *d* is the predator death rate, and *r* is the prey intrinsic growth rate. In this model, predators and prey interact solely through successful predator attacks. Predators cannot grow without reducing prey density; similarly, any reduction in prey populations must contribute to predator population growth.

Both theoretical and empirical studies have challenged ecology's focus on consumption (*a*) in predator-prey dynamics [reviewed in 4,5–8]. The mere threat of predation can be sufficient to reduce prey growth, survival, or fecundity [although the strength of these responses can certainly be affected by factors such as predator hunting mode; 9,10]. Far from being passive players on the ecological stage, prey employ a suite of behavioral and morphological defenses to avoid predation. These defensive strategies often have significant costs that reduce prey fecundity or survival. Predators can thus affect prey populations both by direct consumption (consumptive effects, ‘CE’) and by inducing costly defensive changes in prey traits [nonconsumptive effects, ‘NCE’, also called ‘trait-mediated effects’ by some researchers; 11].

In the past decade, numerous experimental studies have measured the magnitude of NCE, a previously underappreciated class of predator effects. The results have been quite variable: NCE appear to dominate some interactions [e.g., 12], and be weak or absent in others [e.g., 13]. Despite this heterogeneity, it has become clear that NCE are common and that they can have powerful effects on prey populations. A recent meta-analysis found that NCE often rival the effect of CE on prey populations [14]. This result aligns with theoretical predictions [8], [15]–[17] that NCE play an important role in structuring predator-prey interactions, and suggests that the exclusive focus on consumption characteristic of most predator-prey models (Lotka-Volterra and its offshoots) can be misleading.

The integration of NCE into predator-prey theory is clearly critical for progress towards a comprehensive understanding of predator-prey dynamics. We seek to assist such integration by distinguishing among multiple biologically distinct mechanisms by which predator intimidation can affect prey density and, thus, population dynamics. Our hope is that distinguishing among these mechanisms may make it easier to contrast outcomes from different study systems, draw general inferences, and more effectively connect empirical results with predictive models. We develop this framework by systematically expanding the term in the Lotka-Volterra equation through which the effects of predator NCE are manifest, the intrinsic growth rate (*r*). Our intent is not a mathematical analysis of the resulting framework; rather, we examine the different ways in which *r* might respond to predator density, using the Lotka-Volterra model as a heuristic tool for identifying potential NCE pathways and clarifying the relationships between them. Because different types of prey defenses may have distinct ecological effects, genetic causes, and evolutionary consequences, such a classification scheme may help distinguish between biologically distinct types of interactions, allowing a more comprehensive and more mechanistic description of predator-prey dynamics. We integrate our explication of this framework with a metaanalysis of published literature assessing (where possible) the mean effect sizes of both the individual and combined pathways of nonconsumptive predator effects.

## Methods

### Diverse pathways of nonconsumptive predator effects

Different mechanisms of nonconsumptive effects can be categorized by either: 1) the type of trait that is changed in response to predation risk; or 2) the way in which trait changes impact prey population growth. While these two systems may often overlap, they are not identical. We chose the latter approach in this review because it has clear and direct implications for prey (and hence community) dynamics. Traditional Lotka-Volterra equations represent decreases in prey density due to consumption (the term *–aNP* in eq 2). Prey defenses intended to mitigate this mortality rate may incur costs that reduce population growth in other ways, reflected in changes in the population's intrinsic growth rate (*r*) which depend on prey behavior, physiology, and life history. The trade-off between *r* and *a* has been the subject of numerous theoretical studies indicating that prey may optimize their expected fitness by trading off reduced *r* for reduced predation [lower *a*; reviewed in 8,18].

Our approach is to illustrate how *r* may be subdivided into a number of mechanistic variables that can reflect some of the major biologically-distinct pathways by which NCE might arise. This offers an improvement over *r,* which is a phenomenological parameter that cannot provide insight into the pertinent prey traits, or the functional relationship of these traits with predator density and prey growth rates. Rather than provide an exhaustive list, our approach seeks to illustrate the major categories of NCE that have been commonly evaluated by empiricists.

Population growth depends first on the rate of net energy income that can be put towards reproduction (*E_net_*). The net energy income is the difference between the rate of gross energy income (*E_gross_*) and a rate of energetic expenditure necessary for non-reproductive functions (χ, reflecting metabolism, growth, defensive structures, etc.). We further subdivide the gross energy income rate into two components: the foraging effort (*t_f_* , e.g., the proportion of time spent foraging per day), and the energy income per unit foraging time (*E/t*). Equivalently one might measure distance traveled per day while foraging, and energy income per unit distance. Splitting *E_gross_* in this way allows the separate consideration of potentially independent facets of foraging biology that can have distinct ecological impacts (see below). This net rate of energy acquisition,
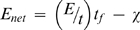
(3)is available for reproduction, with offspring produced at a rate *bE_net_*. The rate of population growth (*r*) is the difference between this birth rate (*bE_net_*) and losses due to a background mortality rate (*m*). If we are dealing with a geographically bounded sub-population rather than an entire species, the local population density may also be modified by immigration (*i*) or emigration rates (*e*). The proportion of individuals available to emigrate is realistically a function of the number of local individuals, whereas the number of immigrants may be density-independent or density-dependent (we assume the former here). We thus substitute the following in place of the intrinsic population growth:
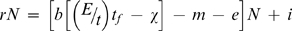
(4)Unlike traditional Lotka-Volterra equations in which *r* is a constant, the terms in equation (4) can be variable. Each term (except *N*) is a function of the prey's behavior, physiology, or biotic or abiotic environment, which in turn depends on the perceived level of risk. Changes in these terms in response to predation risk reduce the attack rate, *a*, but also yield nonconsumptive effects of predators on prey density.

Our development and discussion of the model presented in equation (4) requires several caveats. First, we use our expansion of the Lotka-Volterra equations as a conceptual framework rather than as a mathematical model *per se*; our formulation is thus neither intended nor immediately suitable for dynamical analysis. Such a procedure would require specifying the functional response curves for these different parameters, some of which have been analyzed independently [e.g., foraging effort; 19,20,21] while others have yet to be considered. The model instead provides a heuristic guide to both the major NCE pathways and their relationships, and similar specification for NCEs could be done for any predator-prey model. Second, NCE could also result in changes to other parts of the Lotka-Volterra equation. For instance, NCE that alter prey nutritional value or the efficacy of prey defenses may also affect predator-prey dynamics via changes in the efficiency with which predators convert prey biomass (*e*) or the rate at which prey are successfully attacked (*a*). Predators also modify prey growth rates, and life history schedules, which would have to be incorporated into size or age-structured models. While such feedback processes are clearly important, their detailed explication is beyond the scope of a general review such as ours. Finally, a conceptual framework of this sort necessarily glosses over an array of additional parameters (or further subdivisions of existing ones) that may sometimes prove important. Many of these parameters, and their theoretical underpinnings, have already been reviewed in detail [e.g., 8]; our framework and the accompanying metaanalysis is intended for a broader audience interested in the most widely documented types of NCE pathways, the connections that exist between them, and their relative magnitudes. However, we believe this model-based argument is helpful for explicitly considering different aspects of NCEs. The distinctions between these pathways are often glossed over in empirical studies, most of which are logistically constrained to measuring only one or a few of the mechanisms by which predators might impact prey dynamics.

## Results

### Foraging efficiency, *E/t*

Optimal diet theory suggests that animals adopt foraging strategies intended to maximize some fitness-related currency [reviewed in 22]. The most common assumption is that animals act to maximize the rate of energy income (*E/t*) while foraging. They do so by focusing on the subset of potential food resources providing the highest yield (due to energy content and/or handling time). While optimal diet theory can be successful at predicting foraging behavior [23], many of its more general formulations fail to consider the role of predation risk during foraging. Foraging prey may be less vigilant or more likely to be detected by predators. A prey's optimal diet may therefore not maximize *E/t* once predation risk is accounted for [16], [19], [20].

When energetically profitable habitats are accompanied by a high risk of predation, prey may choose less rewarding (low *E/t*) habitats lower in risk. The tradeoff between optimal foraging effort and risk was formalized by Gilliam and Fraser [22] as the “μ/*f* rule” – prey should forage in habitats with the lowest ratio of mortality (μ) to foraging rate (*f*). For example, juvenile bluegill sunfish avoid predatory largemouth bass by shifting from open-water habitat to safer but energetically less profitable shallow habitats [24]. Grasshoppers similarly avoid spider predation by shifting from highly nutritious grasses to less nutritious forbs [25], [26]. Predators can also reduce energy income rates by modifying foraging behavior within a given habitat. To avoid predators, gray squirrels rejected higher *E/t* food items in favor of less rewarding foods that could be carried to a safe location for consumption [27]. The rate of energy income, *E/t*, may therefore frequently be a negative function of predator density [28].

### Foraging effort, *t_f_*

In addition to reducing the prey's foraging efficiency (energy gained per unit time), predators often reduce the *amount* of time spent foraging per day [reviewed in 29]. While *E/t* and *t_f_* can be combined in models to yield a single rate parameter (*E_gross_*), they are biologically distinct. For instance, the presence of fish predators induces some freshwater snails to spend less time in the water, minimizing their time in aquatic habitats where they feed but are more vulnerable. The resulting decrease in foraging time can substantially affect the snails' fecundity [30], [31]. In principle, the snail's foraging efficiency while in the water may be independent of the length of time spent out of the water avoiding predators. Similarly, mammalian predators such as weasels and stoats intimidate voles into decreasing their foraging time, resulting in lower resource use [32] and reduced growth rates [33]. The fire ant *Solenopsis geminata* stops foraging and adopts a defensive posture in the presence of parasitoid phorid flies [34]. Although the flies cause only low levels of direct mortality (maximum frequency of parasitoid larvae in workers was <3%), they disrupt ant foraging enough to be effective biocontrol agents. Distinguishing between the foraging time and rate terms (*t_f_* and *E/t*) is important because prey reducing one term in response to predation risk may compensate by increasing the other. Furthermore, these two components of foraging behavior may have very different ecological consequences. Reduced foraging on existing resources merely reduces top-down control of the focal species' resources. In contrast, prey may completely switch to another less profitable prey species (changing *E/t* instead of *t_f_*), greatly changing top-down control on the two resource types without affecting the activity cycle of the forager. Consequently, the extent and nature of trophic cascades may greatly depend on the distinction between changes in foraging time versus efficiency.

### Energetic costs, χ

Defenses against predation are not necessarily behavioral; inducible prey responses to predators can include phenotypic changes such as spines, chemical defenses, hormonal stress responses, or autotomy (the intentional loss of a body part in order to avoid predation). These energetically costly defenses often divert resources away from reproduction. Predator-induced defensive morphologies in *Daphnia pulex* are associated with decreases in longevity and clutch size, both of which affect population growth rate [35]. Crest induction in *Daphnia* requires a significant shift of energetic resources away from reproduction. Over a lifetime, crest induction in *Daphnia* required resources equivalent to 60 eggs [36]. Similarly, hoverfly larvae induce a low-fecundity winged morph in their aphid prey [37]. Autotomy can also affect organisms both directly (through the cost of regrowth) and indirectly (through less efficient foraging). Damselfly larvae that dropped their lamellae grew more slowly despite eating the same amount of food, suggesting either that digestive efficiency was reduced, or that regeneration was diverting resources from growth [38]. Stress may also simply lead to an overall increase in metabolic rate [reviewed in 39].

### Effort devoted to reproduction, *b*

Reduced net energy income (a function of the three factors discussed above) can directly impact prey fecundity or survival, constituting a nonconsumptive predator effect on prey demography. However, predators can also reduce prey birth rates independent of their effects on energy income. Attracting a mate often entails high-profile activities that may also increase vulnerability to predators, and several studies have found reductions in mating effort when predation risk is high. Water striders reduce the number and duration of matings in the presence of predatory sunfish [40], while amphipods spend less time in amplexus in lakes with more intense fish predation [41]. Finally, several species of algae reduce recruitment when they detect chemical cues from *Daphnia* herbivores [42]. Long-lived algal species ceased reproduction until the concentration of *Daphnia* cues decrease; the resulting increase in generation time reduces overall population growth rates. In contrast, shorter-lived algal species tested in the same experiment showed no such delay.

The preceding example of delayed reproduction in algae raises a more general issue that neither our Lotka-Volterra formulation nor our across-taxa metaanalysis (below) adequately addresses. Age-structured population models highlight the fact that a population's intrinsic growth rate depends on both the number of progeny and how quickly they are produced. Predation risk can have a number of conflicting effects on prey life history. While higher predation rates may favor faster maturation as prey try to reproduce before being eaten, reduced energy intake due to restricted foraging activity can slow growth rates, resulting in either smaller (and perhaps less fecund) prey, or delayed maturation. For instance, mayfly larvae exposed to a model of their fish predator took 50–80% longer to reach maturity than larvae in a predator-free control [43]. While risk-induced changes in life history clearly affect population growth rates, predation risk may accelerate development in one species and delay it in another. A multi-taxa metaanalysis like ours thus seems unlikely to effectively assess the ‘true’ cost of predation risk on prey development (assessed as time to maturity, length of time per instar, etc.); as a result, we chose not to examine development time *per se* in our meta-analysis.

### Mortality rates not due to consumption, *m*

Predator effects on prey fecundity are compounded by additional non-consumptive effects on prey survival (*m*, equation 6). For instance, non-lethal spiders whose mandibles were glued shut nonetheless had strong starvation-mediated effects on grasshoppers [25]. Grasshoppers that switched from grasses to forbs to minimize predation risk not only grew less, but also had higher mortality rates. While it is counterintuitive that prey would accept starvation over being eaten, such strategies may evolve if a marginal increase in the *probability* of starvation is less than the corresponding marginal decrease in the probability of being captured.

Background mortality rates can depend on predator density in other ways, including failed attacks leading to injury, or increased acceptance of other risk factors. Aphids that drop off host plants to escape predators experience high mortality rates on the ground because of stressful abiotic conditions such as high temperatures [44]. Cues from predatory gastropods induce mussels to close their shells; the mussels are unable to respire with their shells closed and suffer hypoxic conditions that significantly increase their mortality rates [45].

### Immigration and emigration (*e,i*)

Depending on the spatial scale used to define a population, predators can reduce local population density via shifts in the net immigration rate. This behavior has been extensively explored in research involving drift-dispersing aquatic stream invertebrates [reviewed in 46]. Chemical cues from fish or predatory invertebrates induce mayflies and other invertebrate species to increase the rate at which they enter the water column to drift out of local populations. Predator-induced emigration has also been noted in fish [47], marine invertebrates [48], and terrestrial insects [49].

Immigration can also be affected by predator cues. For instance, predatory sunfish with their mouth sewn shut nonetheless reduce hydrophilid beetle densities; predator cues both lower the reproductive activity of resident beetles and reduce the rate at which new beetles colonize the area [50]. While such changes reduce local population densities, they do not necessarily affect the overall metapopulation size of the species or individuals' fitness. However, dispersing animals often experience both energetic costs and increased risks, which may reduce overall population density. A drifting stream invertebrate can forego foraging opportunities, fail to find suitable habitat, or increase the rate at which it encounters other predators. This last possibility highlights the fact that NCE can also occur via interactions with a third species.

Because the effects of migration on prey density are scale-dependent (i.e., local-scale changes in prey density might yield no detectable density differences at the landscape level), it would be inappropriate to calculate a ‘mean’ effect size across studies spanning an array of different spatial scales [51]. This does not mean, however, that local-scale interactions between predators and their prey are not substantively affected by emigration [52]. When the relative effects of consumption and predator-induced emigration on prey density were compared for a series of experimental studies conducted at different spatial scales, emigration appeared to have an equal or greater effect on prey density than consumption for studies of experimental venues ranging from <1 to 35 m in length [53].

### Meta-analysis of NCE pathways

**Methods**: The vast majority of published empirical studies of NCE evaluate only one, or a few, of the preceding pathways. We therefore used meta-analysis to evaluate whether the empirical literature reveals whether some NCE pathways have a consistently large or small effect. We built upon the data set described in Preisser et al. (2005; see this reference for detailed information on search criteria); briefly, we searched for published literature that experimentally assessed the nonconsumptive effect of predators on various prey life-history indices. Because of our interest in the population-level effects of NCE, only papers providing data on one or more of the following prey indices were included in the database: growth (increase in mass or length over time, etc.), fecundity (offspring/female, clutch size, etc.), and survival (% surviving to end of experiment, etc.). All papers fitting the above criteria were also searched for the following indices: activity (% moving per observation, etc.), habitat use (% of time out of shelter, etc.), and feeding rate (resources consumed per unit time, etc.). Data were taken directly from the text or tables, or extracted from graphs or figures. If a study provided data on multiple predator-prey pairs, or on the same predator-prey interaction in substantively different conditions (high versus low nutrient levels, complex versus simple environments, etc.), we treated these studies as independent data points in our analyses in order to address the range of possible conditions in which NCE might be important [e.g., 14,54,55]. If a study examined the same predator-prey pair at different sizes or developmental stages (i.e., a predator's effect on early- versus late-instar prey), we chose the combination with the largest prey/ predator size ratio.

The final 1034-line dataset was generated using 230 papers and included 135 predator species, 179 prey species, and 299 predator-prey species combinations (Appendix S1). There are more entries than there are predator-prey combinations because some pairwise interactions were measured in multiple studies, in multiple contexts (resource levels, predator densities, etc.), or for more than one pathway. The database was heavily weighted towards aquatic systems (963 of 1047 total lines; 92%), leading us to analyze aquatic and terrestrial systems separately. For each line in the dataset we calculated the log response ratio (lnRR) effect size, measured as ln (mean experimental response/mean control response); this procedure is recommended for use with ecological data [56]. We calculated mean effect sizes in terrestrial and aquatic systems using a random-effects model in MetaWin 2.1.4 [57]. While we report mean effect sizes in terrestrial systems for completeness, the relative scarcity of data from these systems suggests that the results of our terrestrial analyses should be treated with caution. We used a bootstrapping routine to calculate 95% confidence intervals around the mean effect size; we chose this procedure to address the fact that the data appeared to be non-normally distributed. Note that, as with any meta-analysis, these estimates represent mean effects that have substantial variance among studies. The precise magnitude of NCE for any of these variables will likely be highly context-dependent even for a given predator-prey species pair. The value of the meta-analysis is to evaluate whether there are aggregate trends across disparate systems and conditions. We calculated Spearman's rank-order correlation, *r_s_*, for each dataset in order to test for potential publication bias [57]–[59].

**Results (** **Table 1** **):** Predator cues strongly affected prey behavioral indices such as feeding rate, overall activity level, and the use of exposed versus sheltered habitats. In aquatic systems, the presence of predator cues reduced prey feeding rate and overall activity by an average of 57% and 42%, respectively (45% and 34% in terrestrial systems). Although indices of prey fitness such as growth and fecundity were less strongly affected, predator cues decreased prey growth and fecundity by an average of 8% and 10% in aquatic systems (9% and 23% in terrestrial systems). Even though it seems unlikely that predation risk alone would increase prey mortality, predator cues decreased prey survival by 5% in aquatic systems (3% in terrestrial systems). All of the preceding trends are significantly different from null hypotheses of no effect. There was no significant difference in effect size between terrestrial and aquatic interactions for any of the six variables (all *p*>0.10). Tests for potential publication bias found no significant correlation between effect size and sample size (*r_s_*, *p*>0.05) in all cases, suggesting that file-drawer effects did not strongly bias our results.

**Table 1:** Mean effect size magnitude of predator risk relative to control treatments in aquatic and terrestrial systems.

			Aquatic Systems		Terrestrial Systems	
Prey Characteristic	Term in Model	Variable(s) assessed in metaanalysis	# interactions (# papers)	mean effect size (95% CI)	# interactions (# papers)	mean effect size (95% CI)
Foraging Efficiency	*E/t*	feeding rate (e.g., resources eaten/time)	52 (22)	0.43 (0.29, 0.60)	8 (7)	0.55 (0.28, 0.98)
Foraging Effort	*t_f_*	activity (e.g., distance moved/time)	149 (50)	0.58 (0.52, 0.65)	10 (8)	0.66 (0.42, 0.96)
Foraging Effort	*t_f_*	habitat choice (e.g., % time in open versus sheltered habitats)	73 (26)	0.63 (0.53, 0.74)	1 (1)	N/A
Net Energy Income	*[(E/t)t_f_ -χ]*	growth rate (e.g., length or biomass increase/time)	491 (147)	0.92 (0.90, 0.94)	27 (17)	0.91 (0.87, 0.95)
Fecundity	*b[(E/t)t_f_ - χ]*	fecundity (e.g., offspring/individual)	118 (42)	0.90 (0.82, 0.99)	25 (15)	0.77 (0.70, 0.85)
Non-consumptive Prey Mortality	*m*	survival (e.g., % prey surviving)	80 (30)	0.95 (0.93, 0.97)	14 (9)	0.97 (0.94, 0.99)

### Beyond 2-species models: Interaction modification

Equations involving only two species cannot fully account for all potential NCE. This is because predators may also modify prey population dynamics by altering the prey's interactions with other species. ‘Interaction modification’ constitutes a major pathway for NCE and includes the consequences of predator-predator interactions and the effect of predator intimidation on interspecific prey competition. In a Lotka-Volterra context, we might say that the loss of prey to a second predator (*−a2PC_2_*) is in part a function of the density of the first predator (*C_1_*).

By inducing defensive phenotypes in their prey, predators can change the rate at which prey are consumed by other predator species [60]. These modifications may be beneficial for the prey (predator-predator inhibition) or detrimental (facilitation). Research by Dayton (1973) illustrates how such interaction modifications relate to NCE. *Pisaster* starfish induce a defensive response in their sea urchin prey: the urchins attempt to move away from the starfish. Because the urchins then have fewer tube feet connected to the substrate, they are more likely to be torn off the rocks by wave action. These displaced urchins are more likely to be eaten by sea anemones [61]. *Pisaster* consequently reduces urchin density both by consuming them, and by increasing the rate at which they fall prey to anemones. These two mortality rates constitute the consumptive and nonconsumptive effects of *Pisaster*, respectively, even though the latter entails a consumptive effect by anemones. While we restrict our attention to predator-predator interactions mediated by prey defensive responses, it is worth noting that direct interactions among predators such as interference competition may have similar effects, and there is some semantic justification for including those as NCE *sensu lato*.

Predator-predator interactions that affect prey survival or population growth rates appear to be widespread. The carnivorous amphipod *Gammarus duebeni* increases the emigration rate of mayfly nymphs, making them more likely to be found and eaten by salmon [62]. Similarly, aphids dropping off their host plant to avoid foliage-dwelling predators are more likely to come into contact with ground-dwelling predators [63]. Predator-predator interactions may not, however, always provide harmful to their prey; egrets, for instance, induce schooling behavior in juvenile spot that reduced their vulnerability to predatory fish [64]. Movements by crayfish can also distract predatory bass, allowing their shared prey, sculpin, to escape [65].

Nonconsumptive predator effects can also arise from multi-species interactions such as interspecific competition [34], [66] or mutualism [67]. When predator-induced behaviors or phenotypes modify the prey's interactions with other species, the predator indirectly affects the prey's population dynamics. Predators that cause their prey to switch to forage in alternative microhabitats are likely to affect the degree to which the prey compete with other species. The interspecific competition term α_1,2_ can thus be viewed as a function of predator threat levels and another potential NCE pathway.

### The value of distinguishing among NCE pathways

The empirical examples described above illustrate a number of biologically distinct routes by which predators can affect prey population growth beyond simple consumption. The classification scheme represented in equation (4) provides a heuristic tool for distinguishing among these mechanisms. We see several advantages to using this scheme as a means of distinguishing among the different mechanisms underlying nonconsumptive predator effects.

First, the mechanisms have different implications for community dynamics and energy flow within a community. Changes in *E/t* are often associated with prey shifting between resources, releasing some resources from consumption while increasing use of others. In contrast, changes in other parameters (e.g., *t_f_*) do not shift the burden of consumption between different species of resources. Hence, NCE involving changes in *E/t* may have fundamentally different community- and ecosystem-level effects than other NCE mechanisms. Changes in *t_f_* reduce the total flow of energy through the prey species, whereas changes in *m*, *e*, *i*, or (*−a_2_PC_2_*) simply redirect where the energy flows. The mechanisms may also require different temporal or spatial scales. Although emigration can almost instantly deplete a local prey population, phenotypic plasticity that redirects energy away from reproduction may have effects that are slow relative to direct consumptive effects.

Phenotypic traits affecting foraging strategies, time allocation, resource allocation, and dispersal behavior may have different genetic architectures, which will affect the likelihood of their evolution. It seems likely that, when faced with a novel predator, prey are more likely to adopt highly plastic behavioral defenses than physiologically based defenses. On the other hand, if physiological defenses have higher heritability than behavioral traits, they may be more readily optimized by natural selection.

Finally, our classification scheme suggests that studies measuring a single NCE pathway will not accurately reflect the actual cumulative interaction strength. Predator-induced decreases in energy income require measurements of both foraging time, and the rate of energy return per unit time. Short-term studies that examine only energetic changes may unwittingly miss the effects of predators on prey fecundity. Field enclosures used to eliminate prey emigration (so that reductions in prey density can be attributed to mortality) also remove an often-significant mechanism by which predators reduce local prey population density. Studies on prey mortality rates may miss effects on fecundity. Finally, changes in one variable may be compensated for by changes in another [68], [69]. We are not aware of any study that has measured all the components of NCE we have described here. Consequently, nearly all studies of NCE (except those of prey population growth rate itself) may significantly under- or over-estimate population level effects of predation risk. While established experimental designs for measuring NCE (e.g., including a ‘non-lethal’ predator treatment alongside control and predator-present manipulations) are an important advance, they are not presently suited for evaluating these multifarious effects of predator intimidation of prey.

## Discussion

We have argued that using Lotka-Volterra equations to address chronic predator effects provides a simple yet fairly general framework for illustrating the fact that predators affect prey dynamics both through consumption (*a*) and by changing prey population growth rates (*r*). These growth rates are in turn a complex function of energy income, energy allocation, mating effort, survivorship, emigration and immigration, all of which depend on prey behavior and physiology. Prey respond to predation risk by changing their behavior and physiology, which in turn can affect population growth rates.

Our metaanalysis confirms that different NCE pathways respond quite differently to predation risk. Behavioral indices such as prey activity, feeding rate, and the use of open versus sheltered habitats were most affected by predator cues, with ∼40–60% changes in the predation risk versus control treatments. The large number of aquatic studies in our dataset, in conjunction with the relatively tight confidence intervals surrounding the mean effect size estimates, argues that our results are likely to be robust. Although we were unable to obtain similar sample sizes for terrestrial systems, there were no significant between-system differences in mean effect size for any of the tested metrices.

The large changes in prey behavior noted above were mirrored by smaller changes in ‘fitness-related’ metrices like growth, fecundity, and even mortality. This finding has several potential (non-exclusive) explanations. First, prey may simply shift their activity budget temporally; predator cues can increase nocturnal activity in a variety of species [e.g., 70]. Second, reductions in one behavior may be offset by increases in another; prey that reduce overall activity levels may, for example, become more efficient at capturing and consuming resources. Third, organisms facing the looming prospect of substantial reductions in growth, fecundity, and survival are likely to eventually increase foraging activity regardless of predation risk [71], [72]. All of the aforementioned mechanisms are consistent with Lima and Bednekoff's risk-allocation hypothesis, which states that, “… the antipredator efforts exhibited in each state of [acute versus chronic] risk are not independent but are inextricably linked.” [p. 650 in 73]. By extension, the trade-offs made by prey are likely to be evolutionarily reasonable in that NCE that negatively affect *r* (e.g., diverting energy from reproduction into chemical or physical defenses) may also positively affect per-capita prey growth through reductions in ‘*a*’ (capture rate). Finally, there is also increasing evidence that some organisms are capable of partially decoupling foraging activity and growth via changes in digestive physiology [68], [69]. Regardless of mechanism, our results suggest that while prey can somewhat compensate for the effects of predation risk, their compensatory response(s) cannot completely buffer the organism against significant decreases in growth, fecundity, and survival.

Despite our increased knowledge of the possible pathways and impacts of NCE, several questions remain unresolved. First, what is the functional relationship between each variable in equation (4) and predation risk? Theoretical treatments of NCE have been predominantly focused on a subset of these variables, and few models have considered the joint action of multiple factors. Complicating the matter, these functional relationships may change with ecological conditions such as resource availability [71]. Second, what is the relative frequency of the various NCE pathways? Are particular NCE pathways generally more likely to evolve in response to predation risk: for example, when should individuals carry out defensive actions that compromise energy gain rather than mating effort? What trade-offs exist between different NCE pathways? Third, how does this multi-faceted view of NCE affect strategies for empirical studies? One concern is that, by focusing on a single aspect of chronic predator effects, studies will tend to under-estimate the total impact of predation risk. On the other hand, the results of our metaanalysis suggest that prey may be capable of compensating for costs at one level by adjusting another trait (e.g., increasing foraging time to compensate for reduced foraging rate). Most studies document a single component of intimidation, or else lump several of them together. For instance, a study showing increased emigration with predation risk tells us nothing about the foraging rates or reproduction of the remaining individuals. Given the apparently widespread effect of NCE, it is important that studies of predator-prey interactions account for their potential fitness and community effects. To the extent to which the diverse mechanisms of these behavioral interactions have different effects, discriminating among them empirically will allow us to better evaluate their consequences.

## Supporting Information

Appendix S1 (0.04 MB PDF)
