# Supplementary material for: The Many Faces of Fear: Comparing the Pathways and Impacts of Nonconsumptive Predator Effects on Prey Populations
Source: PLoS One. 2008 Jun 18;3(6):e2465. doi: 10.1371/journal.pone.0002465 (PMC2409076; doi:10.1371/journal.pone.0002465)
Supplement: Appendix S1 — (0.04 MB PDF) [file pone.0002465.s001.pdf]

Reference	System	Predator species (or closest approximation)	Prey species (or closest approximation)	Response variables measured
Abjornsson et al 2002 Freshwater Biology 47: 1489-1495	Aquatic	Perca fluviatilis, Eurasian perch	non-predatory insects	Growth
Abjornsson et al 2002 Freshwater Biology 47: 1489-1495	Aquatic	Perca fluviatilis, Eurasian perch	predatory insects	Growth
Allouche & Gaudin 2001 Oikos 94: 481-492	Aquatic	Phalacrocorax pygmaeus, pygmy cormorant (stuffed)	Leuciscus cephalus, chub	Activity, Growth
Altwegg 2002 Ecology 83(9): 2542-2551	Aquatic	Anax imperator, dragonfly	Rana lessonae, pool frog	Activity, Growth
Altwegg 2002 Evol Ecol Res 4: 519-536	Aquatic	Anax imperator, dragonfly	Rana esculenta, common European frog	Growth
Altwegg 2002 Evol Ecol Res 4: 519-536	Aquatic	Anax imperator, dragonfly	Rana lessonae, pool frog	Growth
Alvarez & Nicieza 2003 J Fish Biol 63: 1565-1577	Aquatic	Salmo trutta, brown trout	Salmo trutta, brown trout	Habitat use
Alvarez & Peckarsky 2005 Oecologia 142: 576-587	Aquatic	Salvelinus fontinalis, brook trout	Baetis bicaudatus, mayfly	Growth
Anholt et al 2000 Ecology 81(12): 3509-3521	Aquatic	Anax junius, dragonfly	Rana catesbeiana, bullfrog	Growth
Anholt et al 2000 Ecology 81(12): 3509-3521	Aquatic	Anax junius, dragonfly	Rana clamitans, green frog	Growth
Anholt et al 2000 Ecology 81(12): 3509-3521	Aquatic	Anax junius, dragonfly	Rana pipiens, leopard frog	Growth
Anholt et al 2000 Ecology 81(12): 3509-3521	Aquatic	Anax junius, dragonfly	Rana sylvatica, wood frog	Growth
Appleton & Palmer 1988 PNAS 85: 4387-4391	Aquatic	Cancer productus, red rock crab	Nucella lamellosa, gastropod	Growth
Babbitt, K. J. 2001. Can. J. Zoology. 79: 809-814	Aquatic	Anax junius, dragonfly	Rana spenocephala, Southern leopard frog	Activity, Growth
Ball & Baker 1995 Freshwater Biology 34:1-12	Aquatic	Lepomis gibbosus, pumpkinseed sunfish	Chironomus tentans, midge	Growth
Ball & Baker 1996 Ecology 77:1116-1124	Aquatic	Lepomis gibbosus, pumpkinseed sunfish	Chironomus tentans, midge	Growth
Banks & Powell 2004 Oikos 106:176-184	Terrestrial	Vulpes vulpes, red fox	Mus domesticus, house mouse	Fecundity
Barnett & Richardson 2002 Oecologia 132: 436-444	Aquatic	Aeshna palmata, paddle-tailed darner	Rana aurora, red-legged frog	Activity, Growth
Barnett & Richardson 2002 Oecologia 132: 436-444	Aquatic	Aeshna palmata, paddle-tailed darner	Rana pretiosa, Oregon spotted frog	Activity, Growth
Barry 2000 Oecologia 124: 396-401	Aquatic	Anisops gratus, backswimmer	Daphnia carinata	Fecundity, Growth
Barry 2000 Oecologia 124: 396-401	Aquatic	Anisops gratus, backswimmer	Daphnia longicephala	Fecundity, Growth
Barry 2000 Oecologia 124: 396-401	Aquatic	Anisops stali, backswimmer	Daphnia carinata	Fecundity, Growth
Barry 2000 Oecologia 124: 396-401	Aquatic	Anisops stali, backswimmer	Daphnia longicephala	Fecundity, Growth
Beckerman et al 1997 PNAS 94: 10735-10738	Terrestrial	Pisurina mira, nursery-web hunting spider	Melanoplus femurrubrum, red-legged grasshopper	Survival
Belk 1998 Oecologia 113:203-209	Aquatic	Micropterus salmoides, largemouth bass	Lepomis macrochirus, bluegill	Fecundity, Growth, Habitat use
Bernot & Turner 2001 Oecologia 129:139-146	Aquatic	Lepomis gibbosus, pumpkinseed sunfish	Physella integra, snail	Habitat use
Bernot & Turner 2001 Oecologia 129:139-146	Aquatic	Orconectes rusticus, crayfish	Physella integra, snail	Habitat use
Binckley & Resetarits 2003 Oikos 102:623-629	Aquatic	Aphredoderus sayanus, pirate perch	Hyla chrysoscelis, Copes gray treefrog	Fecundity
Binckley & Resetarits 2003 Oikos 102:623-629	Aquatic	Gambusia affinis, western mosquitofish	Hyla chrysoscelis, Copes gray treefrog	Fecundity
Binckley et al 2002 Oecologia 130:157-161	Aquatic	Enneacanthus obesus, banded sunfish	Hyla squirella, squirrel treefrog	Fecundity
Black & Dodson 1990 Oecologia 83: 117-122	Aquatic	Chaoborus americanus, phantom midge	Daphnia pulex	Fecundity, Growth
Black 1993 Limnol Oceanography 38(5): 986-996	Aquatic	Chaoborus americanus, phantom midge	Daphnia pulex	Fecundity, Growth
Black 1993 Limnol Oceanography 38(5): 986-996	Aquatic	Notonecta undulata, backswimmer	Daphnia pulex	Fecundity, Growth
Brodin & Johansson 2002 Oecologia 132:316-322	Aquatic	Perca fluviatilis, Eurasian perch	Lestes sponsa, damselfly	Activity, Growth, Survival
Brodin & Johansson 2004 Ecology 85(11): 2927-2932	Aquatic	Aeshna juncea, dragonfly	Coenagrion hastulatum, damselfly	Activity, Growth
Brodin et al 2006 Oecologia 148: 162-169	Aquatic	Aeshna juncea, dragonfly	Coenagrion hastulatum, damselfly	Activity, Growth, Habitat use
Burks et al 2000 Oikos 88: 139-147	Aquatic	Rutilus rutilus, roach	Daphnia magna	Fecundity, Growth
Caro & Castilla 2004 MEPS 276: 115-123	Aquatic	Acanthocyclus gayi, crab	Semimytilus algosus, mussel	Growth
Caro & Castilla 2004 MEPS 276: 115-123	Aquatic	Concholepas concholepas	Semimytilus algosus, mussel	Growth
Caro & Castilla 2004 MEPS 276: 115-123	Aquatic	Nucella crassilabrum, gastropod	Semimytilus algosus, mussel	Growth
Caudill & Peckarsky 2003 Ecology 84(8): 2133-2144	Aquatic	Salvelinus fontinalis, brook trout	Callibaetis ferrugineus hageni, mayfly	Activity, Growth, Habitat use
Cheung et al 2004 Marine Biology 144: 675-684	Aquatic	Thais clavigera	Perna viridis, green mussel	Growth, Survival
Cheung et al 2004 Marine Biology 144: 675-684	Aquatic	Thalamita danae, swimming crab	Perna viridis, green mussel	Growth, Survival
Chivers et al 1999 J Chem Ecol 25(11): 2455-2463	Aquatic	Notonecta sp., backswimmer	Bufo boreas, Western toad	Growth, Survival
Chivers et al 2001 Oikos 92: 135-142	Aquatic	Desserobdella picta, leech	Hyla regilla, Pacific treefrog	Growth, Survival
Chivers et al 2001 Oikos 92: 135-142	Aquatic	Desserobdella picta, leech	Rana cascadea, Cascades frog	Growth, Survival
Crowder et al 1997 Ecology 78:1796-1804	Aquatic	Paralichthys lethostigma, southern flounder	Leiostomus xanthurus, juvenile spot	Activity
Crowder et al 1997 Ecology 78:1796-1804	Aquatic	wading birds	Leiostomus xanthurus, juvenile spot	Activity
Crowl & Covich 1990 Science 247: 949-951	Aquatic	Orconectes virilis, virile crayfish	Physella virgata virgata, snail	Growth
Dahl & Peckarsky 2003 Oecologia 137:188-94	Aquatic	Rhinichthys cataractae; Etheostoma flabellare	Ephemerella invaria, mayfly	Growth
Danner & Joern 2003 J Insect Behav 16(4): 453-464	Terrestrial	Schizocosa sp., lycosid spider	Ageneotettix deorum, grasshopper	Feeding rate

Reference	System	Predator species (or closest approximation)	Prey species (or closest approximation)	Response variables measured
De Goeij et al 2001 Oecologia 126:500-506	Aquatic	Pleuronectes platessa, plaice (flatfish)	Macoma balthica, tellinid bivalve	Growth
Delgado et al 2002 Biol Bull 203: 112-120	Aquatic	Panulirus argus, spiny lobster	Strombus gigas, queen conch	Activity, Growth
Diehl & Eklov 1995 Ecology 76(6): 1712-1726	Aquatic	Esox lucius, pike	Perca fluviatilis, Eurasian perch	Growth, Habitat use
Diehl & Eklov 1995 Ecology 76(6): 1712-1726	Aquatic	Perca fluviatilis, Eurasian perch	Perca fluviatilis, Eurasian perch	Growth, Habitat use
Dixon & Agarwala 1999 Proc Roy Soc B 266: 1549-1553	Terrestrial	Adalia bipunctata	Acyrthosiphon pisum, green form of pea aphid	Fecundity
Dixon & Agarwala 1999 Proc Roy Soc B 266: 1549-1553	Terrestrial	Adalia bipunctata	Acyrthosiphon pisum, red form of pea aphid	Fecundity
Dixon & Agarwala 1999 Proc Roy Soc B 266: 1549-1553	Terrestrial	Adalia bipunctata	Aphis fabae fabae, black bean aphid	Fecundity
Dixon & Agarwala 1999 Proc Roy Soc B 266: 1549-1553	Terrestrial	Adalia bipunctata	Meguora viciae, vetch aphid	Fecundity
Dixon & Baker 1987 Can J Zool 64:2276-2279	Aquatic	Lepomis gibbosus, pumpkinseed sunfish	Ischnura verticalis, damselfly	Feeding rate
Dodson & Havel 1988 Limnol Ocean 33(6, part 1): 1274-1285	Aquatic	Notonecta undulata, backswimmer	Daphnia pulex	Growth
Downes 2001 Ecology 82(10):2870-2881	Terrestrial	Demansia psammophis, yellow-faced whip snake	Lampropholis guichenoti, garden skink	Activity, Growth
Duvall & Williams 1995 Arch fur Hydrobiol 133(3): 273-286	Aquatic	Oncorhynchus mykiss, rainbow trout	Agnetina capitata, stonefly	Activity, Feeding rate, Growth
Ejdung 1998 MEPS 165: 137-144	Aquatic	Myxocephalus scorpius, short-horned sculpin	Saduria entomon, isopod	Habitat use, Survival
Ejdung 1998 MEPS 165: 137-144	Aquatic	Saduria entomon, isopod	Monoporeia affinis, amphipod	Activity, Survival
Eklov 2000 Oecologia 123:192-199	Aquatic	Anax junius, dragonfly	Rana catesbeiana, bullfrog	Activity, Growth
Eklov 2000 Oecologia 123:192-199	Aquatic	Lepomis macrochirus, bluegill	Rana catesbeiana, bullfrog	Activity, Growth
Feltmate & Williams 1989 Can J Fish Aq Sci 46: 1575-1580	Aquatic	Oncorhynchus mykiss, rainbow trout	Paragnetina media, stonefly	Feeding rate
Fraser & Gilliam 1992 Ecology 73:959-970	Aquatic	Hoplias malabaricus, tiger fish	Rivulus hartii, jumping guabine	Fecundity, Growth
Fuelling & Halle 2004 Oecologia 138: 151-159	Terrestrial	Mustela nivalis nivalis, least weasel	Clethrionomys rufocanus, grey-sided vole	Fecundity
Gliwicz 1994 Oecologia 97: 458-461	Aquatic	Acanthocyclops robustus, copepod	Ceriodaphnia reticulata	Growth
Gliwicz 1994 Oecologia 97: 458-461	Aquatic	Acanthocyclops robustus, copepod	Daphnia hyalina	Growth
Gliwicz 1994 Oecologia 97: 458-461	Aquatic	Acanthocyclops robustus, copepod	Daphnia magna	Growth
Gliwicz 1994 Oecologia 97: 458-461	Aquatic	Acanthocyclops robustus, copepod	Daphnia pulicaria	Growth
Gotceitas 1990 Oecologia 83: 346-351	Aquatic	Micropterus salmoides, largemouth bass	Lepomis macrochirus, bluegill	Feeding rate
Grabowski & Kimbro 2005 Ecology 86(5): 1312-1319	Aquatic	Opsanus tau, oyster toadfish	Panopeus herbstii, mud crab	Habitat use, Survival
Grabowski 2004 Ecology 85(4): 995-1004	Aquatic	Opsanus tau, oyster toadfish	Panopeus herbstii, mud crab	Survival
Grill & Moore 1998 Oecologia 114: 274-282	Terrestrial	simulated	Harmonia axyridis, aposematic ladybird beetle	Growth
Hanazato & Dodson 1992 J Plank Res 14(12): 1743-1755	Aquatic	Chaoborus americanus, phantom midge	Daphnia pulex	Fecundity, Growth
Hanazato 1995 Jpn J Limnol 56(1): 27-32	Aquatic	Lepomis macrochirus, bluegill	Daphnia ambigua	Fecundity, Growth
Hanazato 1995 Jpn J Limnol 56(1): 27-32	Aquatic	Lepomis macrochirus, bluegill	Daphnia galeata	Fecundity, Growth
Harvey 1991 Oecologia 87:29-36	Aquatic	Micropterus salmoides, largemouth bass	Lepomis sp., sunfish	Survival
Havel & Dodson 1987 Hydrobiologia 150: 273-281	Aquatic	Chaoborus americanus, phantom midge	Daphnia pulex	Fecundity, Growth
Heads 1986 Ecol Ent 11: 369-377	Aquatic	Corixa punctata, water boatman	Ischnura elegans, damselfly	Feeding rate
Heads 1986 Ecol Ent 11: 369-377	Aquatic	Leuciscus cephalus, chub	Ischnura elegans, damselfly	Feeding rate
Heads 1986 Ecol Ent 11: 369-377	Aquatic	Notonecta glauca, backswimmer	Ischnura elegans, damselfly	Feeding rate
Hechtel & Juliano 1997 Ecology 78(3): 838-851	Aquatic	Toxorhynchites rutilus, predatory mosquito	Aedes triseriatus, tree-hole mosquito	Activity, Growth
Heikkala et al 1993 Ann Zool Fennici 30:153-161	Terrestrial	Mustela nivalis nivalis, least weasel	Clethrionomys glareolus, bank vole	Growth
Heikkala et al 1993 Ann Zool Fennici 30:153-161	Terrestrial	Mustela nivalis nivalis, least weasel	Clethrionomys rufocanus, grey-sided vole	Growth
Heikkala et al 1993 Ann Zool Fennici 30:153-161	Terrestrial	Mustela nivalis nivalis, least weasel	Clethrionomys rutilus, red vole	Fecundity, Growth
Hellstedt et al 2002 Ann Zool Fenn 39: 257-265	Terrestrial	Mustela nivalis, least weasel	Microtus agrestis, field vole	Activity, Fecundity, Growth
Hill & Lodge 1995 JNABS 14(2): 306-314	Aquatic	Micropterus salmoides, largemouth bass	Orconectes rusticus, O. propinquus, O. virilis	Survival
Hill & Lodge 1999 Ecol Appl 9(2): 678-690	Aquatic	Micropterus salmoides, largemouth bass	Orconectes propinquus, crayfish	Growth, Survival
Hill & Lodge 1999 Ecol Appl 9(2): 678-690	Aquatic	Micropterus salmoides, largemouth bass	Orconectes rusticus, crayfish	Growth, Survival
Hill & Lodge 1999 Ecol Appl 9(2): 678-690	Aquatic	Micropterus salmoides, largemouth bass	Orconectes virilis, virile crayfish	Growth, Survival
Holomuzki & Hatchett 1994 Freshwater Biology 32: 585-592	Aquatic	Lepomis megalotis, longear sunfish	Lirceus fontinalis, isopod	Growth
Huang & Sih 1991 Oecologia 85:530-536	Aquatic	Lepomis cyanellus, green sunfish	Ambystoma barbouri, streamside salamander	Feeding rate, Survival
Jackson & Semlitsch 1993 Ecology 74:342-350	Aquatic	Lepomis macrochirus, bluegill	Ambystoma talpoideum, mole salamander	Growth, Habitat use, Survival
Jeffries 1990 Freshwater Biology 23: 191-196	Aquatic	Aeshna juncea, dragonfly	Enallagma cyathigerum, damselfly	Survival
Jeffries 1990 Freshwater Biology 23: 191-196	Aquatic	Aeshna juncea, dragonfly	Lestes sponsa, damselfly	Survival
Johansson 2002 Can J Zool 80: 944-950	Aquatic	Perca fluviatilis, Eurasian perch	Leucorrhinia dubia, dragonfly	Growth
Johansson et al 2001 Ecology 82(7): 1857-1869	Aquatic	Perca fluviatilis, Eurasian perch	Lestes sponsa, damselfly	Growth

Reference	System	Predator species (or closest approximation)	Prey species (or closest approximation)	Response variables measured
Johnson et al 2003 Can J Zool 81: 1608-1613	Aquatic	Anax junius, dragonfly	Rana spenocephala, Southern leopard frog	Growth
Johnson et al 2003 Can J Zool 81: 1608-1613	Aquatic	Cybister sp., dytiscid beetle	Rana spenocephala, Southern leopard frog	Growth
Johnson et al 2003 Can J Zool 81: 1608-1613	Aquatic	Procambarus nigrocinctus, crayfish	Rana spenocephala, Southern leopard frog	Growth
Jones et al 2003 Oikos 102: 155-163	Aquatic	Lota lota, burbot	Salmo salar, atlantic salmon	Growth
Jones et al 2003 Oikos 102: 155-163	Aquatic	Salmo trutta, brown trout	Salmo salar, atlantic salmon	Growth
Justome et al 1998 Veliger 41(2): 172-179	Aquatic	Leptasteria polaris, starfish	Buccinum undatum, whelk	Activity, Growth
Ketola & Vuorinen 1989 Hydrobiologia 179: 149-155	Aquatic	Chaoborus sp., midge	Daphnia magna	Fecundity
Ketola & Vuorinen 1989 Hydrobiologia 179: 149-155	Aquatic	Chaoborus sp., midge	Daphnia pulex	Fecundity, Growth
Kiesecker et al 2002 J Chem Ecol 28(5): 1017-1015	Aquatic	Taricha granulosa, rough-skinned newt	Rana aurora, red-legged frog	Growth
Klemola et al 1998 Oecologia 115:149-153	Terrestrial	Falco tinnunculus, Eurasian kestrel	Clethrionomys glareolus, bank vole	Fecundity
Klemola et al 1998 Oecologia 115:149-153	Terrestrial	Falco tinnunculus, Eurasian kestrel	Microtus agrestis, field vole	Fecundity
Kohler & McPeek 1989 Ecology 70(6): 1811-1825	Aquatic	Cottus bairdi, mottled sculpin	Baetis tricaudatus, mayfly	Habitat use
Kohler & McPeek 1989 Ecology 70(6): 1811-1825	Aquatic	Cottus bairdi, mottled sculpin	Glossosoma nigrior, caddisfly	Activity
Koskela & Ylonen 1995 Behav Ecol 6(3): 311-315	Terrestrial	Mustela nivalis and Mustela vison	Microtus agrestis, field vole	Activity, Fecundity
Kraft et al 2005 Austral Ecology 30: 558-563	Aquatic	Anax brevistyla, dragonfly	Limnodynastes peronii, striped marsh frog	Growth
Kusch & Chivers 2004 Can. J. Zool. 82: 917-921	Aquatic	Orconectes virilis, virile crayfish	Pimephales promelas, fathead minnow	Growth
LaFiandra & Babbitt 2004 Oecologia 138: 350-359	Aquatic	Anax junius, dragonfly	Hyla femoralis, pinewoods treefrog	Growth, Survival
Lane & Mahoney 2002 J Animal Ecol 71: 780-792	Aquatic	Gambusia holbrookii, mosquitofish	Crinia signifera, eastern brown froglet	Feeding rate, Growth, Survival
Lane & Mahoney 2002 J Animal Ecol 71: 780-792	Aquatic	Gambusia holbrookii, mosquitofish	Limnodynastes tasmaniensis, spotted marsh frog	Feeding rate, Growth, Survival
Langerhans & Dewitt 2002 Evol Ecol Res 6: 857-870	Aquatic	Lepomis gibbosus, pumpkinseed sunfish	Physella virgata, snail	Growth
Lardner 2000 Oikos 88:169-180	Aquatic	Dytiscus marginalis, diving beetle	Bufo bufo, common toad	Growth
Lardner 2000 Oikos 88:169-180	Aquatic	Dytiscus marginalis, diving beetle	Bufo calamita, natterjack toad	Growth
Lardner 2000 Oikos 88:169-180	Aquatic	Dytiscus marginalis, diving beetle	Hyla arborea, common treefrog	Growth
Lardner 2000 Oikos 88:169-180	Aquatic	Dytiscus marginalis, diving beetle	Pelobates fuscus, garlic toad	Growth
Lardner 2000 Oikos 88:169-180	Aquatic	Dytiscus marginalis, diving beetle	Rana arvalis, moor frog	Growth
Lardner 2000 Oikos 88:169-180	Aquatic	Dytiscus marginalis, diving beetle	Rana dalmatina, agile frog	Growth
Lardner 2000 Oikos 88:169-180	Aquatic	Dytiscus marginalis, diving beetle	Rana temporaria, common frog	Growth
Laurila & Kujasalo 1999 J Animal Ecol 68: 1123-1132	Aquatic	Aeshna juncea, dragonfly	Rana temporaria, common frog	Activity, Growth
Laurila et al 1998 Oikos 83: 307-317	Aquatic	Aeshna juncea, dragonfly	Bufo bufo, common toad	Growth, Survival
Laurila et al 1998 Oikos 83: 307-317	Aquatic	Aeshna juncea, dragonfly	Rana temporaria, common frog	Activity, Growth, Survival
Laurila et al 2004 Oikos 107:90-99	Aquatic	Aeshna sp., dragonfly	Rana temporaria, common frog	Activity, Growth
Laurila et al 2006 Oecologia 147(4): 585-595	Aquatic	Aeshna cyanea, dragonfly	Rana arvalis, moor frog	Activity, Growth
Laurila et al 2006 Oecologia 147(4): 585-595	Aquatic	Gasterosteus aculeatus, three-spined stickleback	Rana arvalis, moor frog	Activity, Growth
Laurila et al 2006 Oecologia 147(4): 585-595	Aquatic	Triturus vulgaris, smooth newt	Rana arvalis, moor frog	Activity, Growth
Lefcort et al 1999 Ecol Appl 9(4): 1477-1489	Aquatic	Lepomis macrochirus, bluegill	Lymnaea palustris, snail	Activity
Lefcort et al 1999 Ecol Appl 9(4): 1477-1489	Aquatic	Lepomis macrochirus, bluegill	Rana luteiventris, Columbia spotted frog	Activity
Lewis 2001 Ecology 82(3): 758-765	Aquatic	Orconectes rusticus, crayfish	Amnicola limosa, snail	Growth
Li & Jackson 2005 J Chem Ecol 31(2): 333-342	Terrestrial	Portia labiata, spider-eating jumping spider	Scytodes pallida, egg-carrying spitting spider	Growth, Survival
Li 2002 Proc R Soc Lond Ser B 269: 2155-2161	Terrestrial	Portia labiata, spider-eating jumping spider	Scytodes pallida, egg-carrying spitting spider	Growth, Survival
Lilliendahl 1997 Animal Behav 53: 75-81	Terrestrial	Accipiter nisus, sparrowhawk (stuffed bird)	Carduelis chloris, greenfinch	Activity, Growth
Lilliendahl 1998 Animal Behav 55: 1335-1340	Terrestrial	Accipiter nisus, sparrowhawk (stuffed bird)	Emberiza citrinella, yellowhammer	Growth
Linden et al 2003 Marine Biol 143: 845-850	Aquatic	Perca fluviatilis, Eurasian perch	Neomysis integer, mysid shrimp	Feeding rate, Habitat use
Linden et al 2003 Marine Biol 143: 845-850	Aquatic	Perca fluviatilis, Eurasian perch	Praunus flexuosus, mysid shrimp	Feeding rate, Habitat use
Loose & Dawidowicz 1994 Ecology 75(8): 2255-2263	Aquatic	Leucaspius delineatus, sunbleak	Daphnia magna	Growth
Lopez et al 1995 Rev Chilena Hist Nat 68: 469-475	Aquatic	Nucella crassilabrum, gastropod	Peromytilus purpuratus, mussel	Survival
Luning 1992 Oecologia 92: 383-390	Aquatic	Chaoborus flavicans and Notonecta glauca	Daphnia pulex	Fecundity
Luning 1992 Oecologia 92: 383-390	Aquatic	Chaoborus flavicans, phantom midge	Daphnia pulex	Growth
Luning 1992 Oecologia 92: 383-390	Aquatic	Notonecta glauca, backswimmer	Daphnia pulex	Fecundity, Growth
Luning 1994 Oikos 69: 427-436	Aquatic	Chaoborus flavicans, phantom midge	Daphnia pulex	Fecundity, Growth
Luning 1995 J Plankton Res 17(1): 71-84	Aquatic	Chaoborus flavicans, phantom midge	Daphnia pulex	Fecundity
Macchiusi & Baker 1992 Freshwater Biology 28: 207-216	Aquatic	Lepomis gibbosus, pumpkinseed sunfish	Chironomus tentans, midge	Activity, Growth

Reference	System	Predator species (or closest approximation)	Prey species (or closest approximation)	Response variables measured
Machacek 1993 Limnol Oceanography 38(7): 1544-1550	Aquatic	Rutilus rutilus, roach	Daphnia galeata	Fecundity, Growth
Machacek 1993 Limnol Oceanography 38(7): 1544-1550	Aquatic	Rutilus rutilus, roach	Daphnia obtusa	Fecundity, Growth
Machacek 1995 J Plankton Res 17(7): 1513-1520	Aquatic	Rutilus rutilus, roach	Daphnia galeata	Fecundity, Growth
Magnhagen 1990 Behav Ecol Sociobol 26:331-335	Aquatic	Gadus morhua, cod	Gobus niger, black goby	Fecundity
Magnhagen 1990 Behav Ecol Sociobol 26:331-335	Aquatic	Gadus morhua, cod	Pomatoschistus minutus, sand goby	Fecundity
Mappes et al 1998 Oikos 82: 365-369	Terrestrial	Mustela nivalis nivalis, least weasel	Clethrionomys glareolus, bank vole	Fecundity
Martin & Lopez 1999 Oikos 84: 499-505	Terrestrial	Homo sapiens, an implacable enemy of wall lizards	Podarcis muralis, wall lizard	Habitat use
McCollum & Leimberger 1997 Oecologia 109: 615-621	Aquatic	Anax umbrosa, dragonfly	Hyla chrysoscelis, Copes gray treefrog	Growth
McCollum & Van Buskirk 1996 Evolution 50(2): 583-593	Aquatic	Anax junius, dragonfly	Hyla chrysoscelis, Copes gray treefrog	Activity, Feeding rate, Growth, Survival
McIntosh & Townsend 1996 Oecologia 108: 174-181	Aquatic	Galaxias vulgaris, river galaxia	Deleatidium sp, mayfly	Habitat use
McIntosh & Townsend 1996 Oecologia 108: 174-181	Aquatic	Salmo trutta, brown trout	Deleatidium sp, mayfly	Habitat use
McIntosh et al 2004 Ecology 85(8): 2279-2290	Aquatic	Salvelinus fontinalis, brook trout	Baetis bicaudatus, mayfly	Activity
McIntyre et al 2004 Oecologia 141:130-138	Aquatic	Belostoma malkini, waterbug	Rana palmipes, web-footed frog	Growth, Habitat use, Survival
McNeely et al 1990 Oecologia 85: 69-73	Aquatic	Micropterus dolomieu, smallmouth bass	Orconectes putnami and Cottus bairdi	Habitat use
McPeek et al 2001 Ecology 82(6): 1535-1545	Aquatic	Lepomis gibbosus, pumpkinseed sunfish	Enallagma divagans, damselfly	Growth
McPeek et al 2001 Ecology 82(6): 1535-1545	Aquatic	Lepomis gibbosus, pumpkinseed sunfish	Enallagma laterale, damselfly	Growth
McPeek et al 2001 Ecology 82(6): 1535-1545	Aquatic	Lepomis gibbosus, pumpkinseed sunfish	Ischnura verticalis, damselfly	Growth
Mikolajewski et al 2005 Oikos 110(1): 91-100	Aquatic	Aeshna cyanea, dragonfly	Coenagrion puella, damselfly	Activity, Growth
Moore et al 1996 Oikos 77: 331-335	Aquatic	Lepomis cyanellus, green sunfish	Ambystoma barbouri, streamside salamander	Growth
Morrison 1999 Oecologia 121:113-122	Terrestrial	Pseudacteon browni, phorid fly	Solenopsis geminata, fire ant	Feeding rate
Moses & Sih 1998 Ethology 104:661-669	Aquatic	Notonecta undulata, backswimmer	Gerris marginatus, pond water strider	Activity, Fecundity, Habitat use
Nakaoka 2000 Ecology 81(4):1031-1045	Aquatic	Busycon caria, whelk	Mercenaria mercenaria, clam	Growth
Nicieza 2000 Oecologia 123: 497-505	Aquatic	Salmo salar, atlantic salmon	Rana temporaria, common frog	Growth, Habitat use
Nystrom & Abjornsson 2000 Oikos 88:181-190	Aquatic	Oncorhynchus mykiss, rainbow trout	Bufo bufo, common toad	Habitat use, Survival
Nystrom & Abjornsson 2000 Oikos 88:181-190	Aquatic	Oncorhynchus mykiss, rainbow trout	Rana temporaria, common frog	Habitat use, Survival
Oku et al 2004 J Ethology 22:109-112	Terrestrial	Amblyseius womersleyi, predatory mite	Tetranychus kanzawai, phytophagous mite	Fecundity
Orizaola & Brana 2005 Freshwater Biology 50:438-446	Aquatic	Salmo trutta, brown trout	Triturus helveticus, palmate newt	Growth
Oriziola and Brana 2004 Ann. Zool. Fennici 41:635-645	Aquatic	Salmo trutta, brown trout	Triturus alpestris, alpine newt	Growth, Survival
Oriziola and Brana 2004 Ann. Zool. Fennici 41:635-645	Aquatic	Salmo trutta, brown trout	Triturus boscai, newt	Growth, Survival
Oriziola and Brana 2004 Ann. Zool. Fennici 41:635-645	Aquatic	Salmo trutta, brown trout	Triturus helveticus, palmate newt	Growth, Survival
Oriziola and Brana 2004 Ann. Zool. Fennici 41:635-645	Aquatic	Salmo trutta, brown trout	Triturus marmoratus, newt	Growth, Survival
Palmer 1990 Hydrobiologia 193: 155-182	Aquatic	Cancer pagurus, crab	Nucella lapillus, Atlantic dogwhelk	Growth
Peacor & Werner 1997 Ecology 78:1446-1156	Aquatic	Anax longipes, comet darner	Rana catesbeiana, bullfrog	Growth
Peacor & Werner 1997 Ecology 78:1446-1156	Aquatic	Anax longipes, comet darner	Rana clamitans, green frog	Growth
Peacor & Werner 2000 Ecology 81(7):1998-2010	Aquatic	Anax longipes, comet darner	Rana catesbeiana, bullfrog	Growth
Peacor & Werner 2000 Ecology 81(7):1998-2010	Aquatic	Anax longipes, comet darner	Rana clamitans, green frog	Growth
Peacor & Werner 2004 Is. J. Zool 50:139-167	Aquatic	Anax junius, dragonfly	Rana sylvatica, wood frog	Growth
Peacor 2002 Ecology Letters 5:77-85	Aquatic	Anax sp., dragonfly	Rana catesbeiana, bullfrog	Activity, Growth
Peckarsky 1996 Ecology 77(6): 1888-1905	Aquatic	Kogotus modestus, stonefly	Baetis bicaudatus, mayfly	Habitat use
Peckarsky 1996 Ecology 77(6): 1888-1905	Aquatic	Kogotus modestus, stonefly	Cinygmula sp., mayfly	Feeding rate
Peckarsky 1996 Ecology 77(6): 1888-1905	Aquatic	Kogotus modestus, stonefly	Epeorus deceptivus, mayfly	Feeding rate, Habitat use
Peckarsky 1996 Ecology 77(6): 1888-1905	Aquatic	Megarcys signata, stonefly	Baetis bicaudatus, mayfly	Habitat use
Peckarsky 1996 Ecology 77(6): 1888-1905	Aquatic	Megarcys signata, stonefly	Cinygmula sp., mayfly	Feeding rate
Peckarsky 1996 Ecology 77(6): 1888-1905	Aquatic	Megarcys signata, stonefly	Epeorus longimanus, mayfly	Feeding rate, Habitat use
Peckarsky 1996 Ecology 77(6): 1888-1905	Aquatic	Megarcys signata, stonefly	Ephemerella sp., mayfly	Feeding rate
Peckarsky et al 1993 Ecology 74:1836-1846	Aquatic	Kogotus modestus, stonefly	Baetis bicaudatus, mayfly	Feeding rate
Peckarsky et al 1993 Ecology 74:1836-1846	Aquatic	Megarcys signata, stonefly	Baetis bicaudatus, mayfly	Fecundity, Feeding rate, Growth
Peckarsky et al 2002 Ecology 83(3): 612-618	Aquatic	Salvelinus fontinalis, brook trout	Baetis bicaudatus, mayfly	Growth
Persons et al 2002 Behav Ecol 13(3): 386-392	Terrestrial	Hogna helluo, wolf spider	Pardosa milvina, wolf spider	Fecundity, Survival
Petranka & Fakhoury 1991 Copeia 1: 234-239	Aquatic	Lepomis macrochirus, bluegill	Anopheles sp., mosquito	Fecundity
Petranka & Fakhoury 1991 Copeia 1: 234-239	Aquatic	Lepomis macrochirus, bluegill	Chaoborus sp., midge	Fecundity

Reference	System	Predator species (or closest approximation)	Prey species (or closest approximation)	Response variables measured
Pierce 1988 Oecologia 77: 81-90	Aquatic	Lepomis macrochirus, bluegill	Ladona deplanata, dragonfly	Habitat use
Pierce 1988 Oecologia 77: 81-90	Aquatic	Lepomis macrochirus, bluegill	Sympetrum semicinctum, dragonfly	Habitat use
Pierce 1988 Oecologia 77: 81-90	Aquatic	Lepomis macrochirus, bluegill	Tetragoneuria cynosura, dragonfly	Habitat use
Pravosudov & Grubb 1998 Animal Behav 56: 49-54	Terrestrial	Accipiter striatus, sharp-shinned hawk	Baelophus bicolor, tufted titmouse	Growth
Pusenius & Ostfeld 2000 Oikos 91(1): 123-130	Terrestrial	Mustela erminea, stoat	Microtus pennsylvanicus, meadow vole	Survival
Pusenius & Ostfeld 2002 Ecography 25: 481-87	Terrestrial	urine/feces of Vulpes vulpes, Canis latrans, Felix rufus	Microtus pennsylvanicus, meadow vole	Feeding rate, Survival
Rahel & Stein 1988 Oecologia 75:9498	Aquatic	Micropterus dolomieu, smallmouth bass	Etheostoma nigrum, johnny darter	Habitat use
Rahel & Stein 1988 Oecologia 75:9498	Aquatic	Orconectes rusticus, crayfish	Etheostoma nigrum, johnny darter	Habitat use
Rasmy et al 1990 Expt. & Appl. Acarology 10: 151-155	Terrestrial	Amblyseius gossipi, predatory mite	Tetranychus urticae, two-spotted spider mite	Fecundity
Rasmy et al 1990 Expt. & Appl. Acarology 10: 151-155	Terrestrial	Phytoseiulus finitimus, predatory mite	Tetranychus urticae, two-spotted spider mite	Fecundity
Rasmy et al 1990 Expt. & Appl. Acarology 10: 151-155	Terrestrial	Phytoseiulus persimilis, predatory mite	Tetranychus urticae, two-spotted spider mite	Fecundity
Rawlings 1994 JEMBE 181: 67-79	Aquatic	Cancer productus, red rock crab	Nucella emarginata, snail	Fecundity, Growth
Reede & Ringelberg 1995 Hydrobiologia 307: 207-212	Aquatic	Perca fluviatilis, Eurasian perch	Daphnia hyalina x galeata	Growth
Reede 1995 J Plankton Res 17(8): 1661-1667	Aquatic	Perca fluviatilis, Eurasian perch	Daphnia hyalina x galeata	Fecundity, Growth, Survival
Reede 1997 Freshwater Biol 37: 389-396	Aquatic	Perca fluviatilis, Eurasian perch	Daphnia hyalina x galeata	Fecundity, Growth
Reimer & Harms-Ringdahl 2001 Mar Biol 139: 959-965	Aquatic	Asterias rubens, starfish	Mytilus edulis, blue mussel	Growth
Reimer & Harms-Ringdahl 2001 Mar Biol 139: 959-965	Aquatic	Carcinus maenas, green crab	Mytilus edulis, blue mussel	Growth
Reimer & Tedergren 1996 Oikos 75: 383-390	Aquatic	Asterias rubens, starfish	Mytilus edulis, blue mussel	Growth
Reimer et al 1995 Mar Fresh Behav Physiol 25: 233-244	Aquatic	Asterias rubens, starfish	Mytilus edulis, blue mussel	Feeding rate, Growth
Relyea & Hoverman 2003 Oecologia 134:596-604.	Aquatic	Anax sp., dragonfly	Hyla versicolor, gray treefrog	Growth
Relyea & Werner 1999 Ecology 80(6): 2117-2124	Aquatic	Anax sp., dragonfly	Rana catesbeiana, bullfrog	Activity, Growth
Relyea & Werner 1999 Ecology 80(6): 2117-2124	Aquatic	Anax sp., dragonfly	Rana clamitans, green frog	Activity, Growth
Relyea & Werner 1999 Ecology 80(6): 2117-2124	Aquatic	Lepomis macrochirus, bluegill	Rana catesbeiana, bullfrog	Activity, Growth
Relyea & Werner 1999 Ecology 80(6): 2117-2124	Aquatic	Lepomis macrochirus, bluegill	Rana clamitans, green frog	Activity, Growth
Relyea & Werner 1999 Ecology 80(6): 2117-2124	Aquatic	Umbra limi, mudminnow	Rana catesbeiana, bullfrog	Activity, Growth
Relyea & Werner 1999 Ecology 80(6): 2117-2124	Aquatic	Umbra limi, mudminnow	Rana clamitans, green frog	Activity, Growth
Relyea & Werner 2000 Copeia 2000(1): 178-190	Aquatic	Anax sp., dragonfly	Rana pipiens, leopard frog	Growth
Relyea & Yurewicz 2002 Oecologia 131:569-579.	Aquatic	Ambystoma tigrinum, tiger salamander	Rana clamitans, green frog	Growth, Survival
Relyea & Yurewicz 2002 Oecologia 131:569-579.	Aquatic	Anax sp., dragonfly	Rana clamitans, green frog	Growth, Survival
Relyea & Yurewicz 2002 Oecologia 131:569-579.	Aquatic	Anax sp., dragonfly, and Ambystoma tigrinum	Rana clamitans, green frog	Growth, Survival
Relyea 2000 Ecology 81(8): 2278-2289	Aquatic	Anax sp., dragonfly	Rana pipiens, leopard frog	Activity, Growth
Relyea 2000 Ecology 81(8): 2278-2289	Aquatic	Anax sp., dragonfly	Rana sylvatica, wood frog	Activity, Growth
Relyea 2000 Ecology 81(8): 2278-2289	Aquatic	Umbra limi, mudminnow	Rana pipiens, leopard frog	Activity, Growth
Relyea 2000 Ecology 81(8): 2278-2289	Aquatic	Umbra limi, mudminnow	Rana sylvatica, wood frog	Activity, Growth
Relyea 2002 Am Nat 159(3): 272-282	Aquatic	Anax longipes, comet darner	Rana sylvatica, wood frog	Activity, Growth
Relyea 2002 Ecol Mon 72(1): 77-93	Aquatic	Anax sp., dragonfly	Rana sylvatica, wood frog	Activity, Growth
Relyea 2002 Ecol Mon 72(4): 523-540	Aquatic	Anax longipes, comet darner	Rana sylvatica, wood frog	Activity, Growth
Relyea 2002 Ecology 83(7): 1953-1964	Aquatic	Anax longipes, comet darner	Hyla versicolor, gray treefrog	Activity, Growth, Survival
Relyea 2003 Ecology 84(7): 1827-1839	Aquatic	Anax sp., dragonfly	Rana sylvatica, wood frog	Activity, Growth
Relyea 2003 Ecology 84(7): 1827-1839	Aquatic	Belostoma sp., waterbug	Rana sylvatica, wood frog	Activity, Growth
Relyea 2003 Ecology 84(7): 1827-1839	Aquatic	Dytiscus sp., diving beetle	Rana sylvatica, wood frog	Activity, Growth
Relyea 2003 Ecology 84(7): 1827-1839	Aquatic	Erythemis sp., dragonfly	Rana sylvatica, wood frog	Activity, Growth
Relyea 2004 Ecology 85(1): 172-179	Aquatic	Anax junius, dragonfly	Rana sylvatica, wood frog	Growth
Repka & Pihlajamaa 1996 Hydrobiologia 339: 67-71	Aquatic	Chaoborus obscuripes, midge	Daphnia pulex	Fecundity, Growth
Repka et al 1994 Hydrobiologia 294: 129-140	Aquatic	Chaoborus obscuripes, midge	Daphnia pulex	Fecundity, Growth
Repka et al 1994 Hydrobiologia 294: 129-140	Aquatic	Dytiscus sp., diving beetle	Daphnia pulex	Fecundity, Growth
Repka et al 1994 Hydrobiologia 294: 129-140	Aquatic	Mochlonyx sp., midge	Daphnia pulex	Fecundity, Growth
Repka et al 1994 Hydrobiologia 294: 129-140	Aquatic	Notonecta sp., backswimmer	Daphnia pulex	Fecundity, Growth
Resetarits 2001 Oecologia 129:155-160	Aquatic	Lepomis gibbosus, pumpkinseed sunfish	Tropisternus lateralis, hydrophilid beetle	Fecundity
Resetarits 2005 Ecology Letters 8: 480-486	Aquatic	Enneacanthus obesus, banded sunfish	Hyla chrysoscelis, Copes gray treefrog	Fecundity
Resetarits et al 2004 Oecologia 138:532-538	Aquatic	Enneacanthus obesus, banded sunfish	Hyla chrysoscelis, Copes gray treefrog	Growth, Survival

Reference	System	Predator species (or closest approximation)	Prey species (or closest approximation)	Response variables measured
Richardson & Brown 1992 JEMBE 163: 169-182	Aquatic	Menippe adina, stone crab	Thais haemastoma, rock snail	Survival
Rieger et al 2004 Ecology 85(8): 2094-2099	Aquatic	Umbra pygmaea, Eastern mudminnow	Hyla femoralis, pinewoods treefrog	Fecundity
Roitberg et al 1979 J Animal Ecol 48:111-122	Terrestrial	Coccinella californica, ladybird beetle	Acyrthosiphon pisum, pea aphid	Survival
Ronkainen & Ylonen 1994 Oecologia 97: 377-381	Terrestrial	Mustela erminea, stoat	Clethrionomys glareolus, bank vole	Activity
Saenz et al 2003 Copeia 3: 646-649	Aquatic	Procambarus nigrocinctus, crayfish	Rana spenocephala, Southern leopard frog	Survival
Schaffner & Anholt 1998 J Insect Behav 11(6): 793-809	Aquatic	Anax imperator, dragonfly	Ischnura elegans, damselfly	Activity, Growth
Schalk et al 2002 Copeia 2002(2): 445-449	Aquatic	Macrobdella decora, leech	Rana clamitans, green frog	Growth
Scheiner & Berrigan 1998 Evolution 52(2): 368-378	Aquatic	Chaoborus americanus, phantom midge	Daphnia pulex	Fecundity, Growth
Scheuerlein et al 2001 Proc Roy Soc B 268: 1575-1582	Terrestrial	Lanius collaris, fiscal shrike	Saxicolla toquata axillaris, Stonechat	Growth
Schmidt & Van Buskirk 2005 J Evol Biol 18: 415-425	Aquatic	Aeshna cyanea, dragonfly	Triturus carnifex, newt	Activity, Growth
Schmidt & Van Buskirk 2005 J Evol Biol 18: 415-425	Aquatic	Aeshna cyanea, dragonfly	Triturus cristatus, newt	Activity, Growth
Schmidt & Van Buskirk 2005 J Evol Biol 18: 415-425	Aquatic	Aeshna cyanea, dragonfly	Triturus marmoratus, newt	Activity, Growth
Schmidt & Van Buskirk 2005 J Evol Biol 18: 415-425	Aquatic	Aeshna cyanea, dragonfly	Triturus vulgaris, smooth newt	Activity, Growth
Schmitz Am Nat 1998 151(4): 327-342	Terrestrial	Pisurina mira, nursery-web hunting spider	Chorthippus curtipennis, grasshopper	Survival
Schmitz Am Nat 1998 151(4): 327-342	Terrestrial	Pisurina mira, nursery-web hunting spider	Melanoplus femurrubrum, red-legged grasshopper	Survival
Schmitz et al. Ecology 1997 78(5): 1388-1399	Terrestrial	Pisurina mira, nursery-web hunting spider	Melanoplus femurrubrum, red-legged grasshopper	Activity, Survival
Schoeppner & Relyea 2005 Ecology Letters 8: 505-512	Aquatic	Anax junius, dragonfly	Hyla versicolor, gray treefrog	Activity, Growth
Scrimgeour & Culp 1994 Oecologia 100:128-134	Aquatic	Rhinichthys cataractae, longnose dace	Baetis tricaudatus, mayfly	Fecundity, Growth
Sih & Krupa 1996 Oecologia 105:179-188	Aquatic	Lepomis cyanellus, green sunfish	Aquarius remigis, stream waterstrider	Fecundity
Sih et al 1990 Am. Nat. 135:284-290	Aquatic	Lepomis cyanellus, green sunfish	Gerris remigis, water strider	Activity, Fecundity
Skelly 1992 Ecology 73(2): 704-708	Aquatic	Ambystoma tigrinum tigrinum, tiger salamander	Hyla versicolor, gray treefrog	Activity, Growth
Skelly 1995 Ecology 76(1):150-164	Aquatic	Anax junius, dragonfly	Pseudacris crucifer, spring peeper	Activity, Growth
Skelly 1995 Ecology 76(1):150-164	Aquatic	Anax junius, dragonfly	Pseudacris triseriata, chorus frog	Activity, Growth
Skelly and Werner 1990 Ecology 71(6): 2313-2322	Aquatic	Anax junius, dragonfly	Bufo americanus, american toad	Activity, Growth
Smith & Jennings 2000 Mar Biol 136: 461-469	Aquatic	Carcinus maenas, green crab	Mytilus edulis, blue mussel	Growth
Smith & Jennings 2000 Mar Biol 136: 461-469	Aquatic	Nucella lapillus, Atlantic dogwhelk	Mytilus edulis, blue mussel	Growth
Soluk & Collins 1988 Oikos 52:94-100	Aquatic	Cottus bairdi, mottled sculpin	Agnetina capitata, stonefly	Survival
Sparrevik and Leonardsson 1999 Oecologia 120: 77-86	Aquatic	Saduria entomon, isopod	Monoporeia affinis, amphipod	Activity, Fecundity, Growth
Stachowicz & Hay 1999 Ecology 80(2): 495-509	Aquatic	Lagodon rhomboides, pinfish	Libinia dubia, decorator crab	Feeding rate
Stamp & Bowers 1991 Oecologia 88: 325-330	Terrestrial	Podisus maculiventris, stinkbug	Junonia coenia, common buckeye butterfly	Survival
Stamp & Bowers 1991 Oecologia 88: 325-330	Terrestrial	Polistes dominulus and P. fuscatus, vespid wasps	Hemileuca lucina, New England buckmoth	Survival
Stamp & Bowers 1993 Oecologia 95: 376-384	Terrestrial	Podisus maculiventris, stinkbug	Junonia coenia, common buckeye butterfly	Survival
Stamp & Bowers 1993 Oecologia 95: 376-384	Terrestrial	Polistes fuscatus, wasp	Junonia coenia, common buckeye butterfly	Survival
Stamp 1997 Oikos 79:147-154	Terrestrial	Polistes fuscatus, wasp (simulated)	Junonia coenia, common buckeye butterfly	Growth
Stamp 1997 Oikos 79:147-154	Terrestrial	Polistes fuscatus, wasp (simulated)	Pyrrharctia isabella, Isabella tiger moth	Growth
Stemberger 1988 Limnol Ocean 33(4, part 1): 593-606	Aquatic	Asplanchna brightwelli, rotifer	Keratella testudo, rotifer	Growth
Stibor & Luning 1994 Functional Ecology 8: 97-101	Aquatic	Chaoborus flavicans, phantom midge	Daphnia hyalina	Growth
Stibor & Luning 1994 Functional Ecology 8: 97-101	Aquatic	Leuciscus idus, golden orfe	Daphnia hyalina	Growth
Stibor & Luning 1994 Functional Ecology 8: 97-101	Aquatic	Notonecta glauca, backswimmer	Daphnia hyalina	Growth
Stibor 1992 Oecologia 92: 162-165	Aquatic	Leuciscus idus, golden orfe	Daphnia hyalina	Fecundity
Stoks & McPeek 2003 Ecology 84(12): 3327-3338	Aquatic	Anax junius, dragonfly	Lestes congener, damselfly	Activity, Habitat use
Stoks & McPeek 2003 Ecology 84(12): 3327-3338	Aquatic	Anax junius, dragonfly	Lestes disjunctus, damselfly	Activity, Habitat use
Stoks & McPeek 2003 Ecology 84(12): 3327-3338	Aquatic	Anax junius, dragonfly	Lestes dryas, damselfly	Activity, Growth, Habitat use
Stoks & McPeek 2003 Ecology 84(12): 3327-3338	Aquatic	Anax junius, dragonfly	Lestes eurinus, damselfly	Activity, Growth, Habitat use
Stoks & McPeek 2003 Ecology 84(12): 3327-3338	Aquatic	Anax junius, dragonfly	Lestes forcipatus, damselfly	Activity, Habitat use
Stoks & McPeek 2003 Ecology 84(12): 3327-3338	Aquatic	Anax junius, dragonfly	Lestes rectangularis, damselfly	Activity, Growth, Habitat use
Stoks & McPeek 2003 Ecology 84(12): 3327-3338	Aquatic	Anax junius, dragonfly	Lestes vigilax, damselfly	Activity, Growth, Habitat use
Stoks & McPeek 2003 Ecology 84(12): 3327-3338	Aquatic	Lepomis gibbosus, pumpkinseed sunfish	Lestes congener, damselfly	Activity, Habitat use
Stoks & McPeek 2003 Ecology 84(12): 3327-3338	Aquatic	Lepomis gibbosus, pumpkinseed sunfish	Lestes disjunctus, damselfly	Activity, Habitat use
Stoks & McPeek 2003 Ecology 84(12): 3327-3338	Aquatic	Lepomis gibbosus, pumpkinseed sunfish	Lestes dryas, damselfly	Activity, Growth, Habitat use
Stoks & McPeek 2003 Ecology 84(12): 3327-3338	Aquatic	Lepomis gibbosus, pumpkinseed sunfish	Lestes eurinus, damselfly	Activity, Growth, Habitat use

Reference	System	Predator species (or closest approximation)	Prey species (or closest approximation)	Response variables measured
Stoks & McPeek 2003 Ecology 84(12): 3327-3338	Aquatic	Lepomis gibbosus, pumpkinseed sunfish	Lestes forcipatus, damselfly	Activity, Habitat use
Stoks & McPeek 2003 Ecology 84(12): 3327-3338	Aquatic	Lepomis gibbosus, pumpkinseed sunfish	Lestes rectangularis, damselfly	Activity, Growth, Habitat use
Stoks & McPeek 2003 Ecology 84(12): 3327-3338	Aquatic	Lepomis gibbosus, pumpkinseed sunfish	Lestes vigilax, damselfly	Activity, Growth, Habitat use
Stoks 1998 Oecologia 117:443-448	Aquatic	Notonecta glauca, backswimmer	Lestes sponsa, damselfly	Activity, Feeding rate
Stoks 2001 Oecologia 127:222-229	Aquatic	Aeshna cyanea, dragonfly	Lestes sponsa, damselfly	Growth
Stoks et al 1999 Evol Ecol 13: 115-129	Aquatic	Aeshna cyanea, dragonfly	Lestes sponsa, damselfly	Growth, Survival
Stoks et al 1999 Oecologia 120:87-91	Aquatic	Aeshna cyanea, dragonfly	Lestes sponsa, damselfly	Growth, Survival
Stoks et al 2005 J Animal Ecol 74: 708-715	Aquatic	Perca fluviatilis, Eurasian perch	Lestes sponsa, damselfly	Growth
Storfer & Sih 1998 Evolution 52(2): 558-565	Aquatic	Lepomis cyanellus, green sunfish	Ambystoma barbouri, streamside salamander	Feeding rate
Storfer & White 2005 J Herp 38(4): 612-615	Aquatic	Anax junius, dragonfly	Ambystoma tigrinum nebulosum	Growth
Storfer & White 2005 J Herp 38(4): 612-615	Aquatic	Dytiscus sp., diving beetle	Ambystoma tigrinum nebulosum	Growth
Takahara et al 2003 Ecological Res 18: 793-806	Aquatic	Carassius auratus, goldfish	Hyla japonica, treefrog	Survival
Teplitsky et al 2004 Ecology 85(10):2888-2894	Aquatic	Aeshna cyanea, dragonfly	Rana dalmatina, agile frog	Growth
Teplitsky et al 2004 Ecology 85(10):2888-2894	Aquatic	Aeshna cyanea, dragonfly	Rana ridibunda	Growth
Teplitsky et al 2004 Ecology 85(10):2888-2894	Aquatic	Gasterosteus aculeatus, three-spined stickleback	Rana dalmatina, agile frog	Growth
Teplitsky et al 2004 Ecology 85(10):2888-2894	Aquatic	Gasterosteus aculeatus, three-spined stickleback	Rana ridibunda	Growth
Teplitsky et al 2005 Oecologia 145: 364-370	Aquatic	Gasterosteus aculeatus, three-spined stickleback	Rana dalmatina, agile frog	Growth
Thiemann & Wassersug 2000 Biol J Linn Soc 71: 513-528	Aquatic	Fundulus diaphanus, banded killifish	Rana clamitans, green frog	Activity, Growth
Tollrian 1995 Ecology 76(6): 1691-1705	Aquatic	Chaoborus flavicans, phantom midge	Daphnia pulex	Fecundity, Growth
Trussell & Nicklin 2002 Ecology 83(6): 1635-1647	Aquatic	Carcinus maenas, green crab	Littorina obtusata, snail	Growth
Trussell & Smith 2000 PNAS 97(5): 2123-2127	Aquatic	Carcinus maenas, green crab	Littorina obtusata, snail	Growth
Trussell et al 2003 Ecology 84(3): 629-640	Aquatic	Carcinus maenas, green crab	Littorina littorea, snail	Growth
Trussell et al 2003 Ecology 84(3): 629-640	Aquatic	Carcinus maenas, green crab	Nucella lapillus, Atlantic dogwhelk	Growth
Turner & Montgomery 2003 Ecology 84(3): 616-622	Aquatic	Lepomis gibbosus, pumpkinseed sunfish	Physa acuta, snail	Growth, Habitat use
Turner 2004 Oikos 104(3): 561-569	Aquatic	Cambarus bartonii, crayfish	Helisoma trivolvis, pulmonate snail	Growth, Habitat use
Turner et al 2000 Oikos 88:148-158	Aquatic	Lepomis gibbosus, pumpkinseed sunfish	Physella gyrina, freshwater snail	Habitat use
Van Buskirk & Schmidt 2000 Ecology 81(11): 3009-3028	Aquatic	Aeshna cyanea, dragonfly	Triturus alpestris, alpine newt	Habitat use, Survival
Van Buskirk & Schmidt 2000 Ecology 81(11): 3009-3028	Aquatic	Aeshna cyanea, dragonfly	Triturus helveticus, palmate newt	Growth, Habitat use, Survival
Van Buskirk and Yurewicz 1998 Oikos 82: 20-28	Aquatic	Anax junius, dragonfly	Rana sylvatica, wood frog	Activity, Growth
Vorndran et al 2002 Ecology 83(6): 1648-1659	Aquatic	Aeshna cyanea, dragonfly	Bombina bombina, fire-bellied toad	Growth
Vorndran et al 2002 Ecology 83(6): 1648-1659	Aquatic	Aeshna cyanea, dragonfly	Bombina variegata, fire-bellied toad	Growth
Walker & Rypstra 2003 J Arachnology 31: 425-427	Terrestrial	Hogna helluo, wolf spider	Pardosa milvina, wolf spider	Survival
Walls et al 1991 Oecologia 87:43-50	Aquatic	Chaoborus crystallinus, midge	Daphnia pulex	Fecundity, Survival
Walls et al 1997 Freshwater Biology 38:353-364	Aquatic	Chaoborus sp., midge	Daphnia pulex	Growth
Walls et al 2002 Herpetologica 58(1):104-118	Aquatic	Gambusia affinis, western mosquitofish	Gastrophryne carolinensis, narrow-mouthed toad	Growth, Habitat use, Survival
Walls et al 2002 Herpetologica 58(1):104-118	Aquatic	Gambusia affinis, western mosquitofish	Hyla squirella, squirrel treefrog	Growth, Habitat use, Survival
Walls et al 2002 Herpetologica 58(1):104-118	Aquatic	Procambarus sp., crayfish	Gastrophryne carolinensis, narrow-mouthed toad	Growth, Survival
Weber & Declerk 1997 Hydrobiologia 360: 89-99	Aquatic	Chaoborus americanus, phantom midge	Daphnia galeata	Fecundity, Growth, Survival
Weber & Declerk 1997 Hydrobiologia 360: 89-99	Aquatic	Perca fluviatilis, Eurasian perch	Daphnia galeata	Fecundity, Growth, Survival
Weber 2001 J Plankton Research 23(1):41-46	Aquatic	Chaoborus sp., midge	Daphnia galeata	Growth
Weber 2001 J Plankton Research 23(1):41-46	Aquatic	Perca fluviatilis, Eurasian perch	Daphnia galeata	Growth
Weber et al 2003 Hydrobiologia 491: 273-287	Aquatic	Chaoborus americanus, phantom midge	Daphnia galeata	Growth
Weetman & Atkinson 2002 Oikos 98: 299-307	Aquatic	Gasterosteus aculeatus, three-spined stickleback	Daphnia pulex	Fecundity
Werner & Anholt 1996 Ecology 77:157-169	Aquatic	Anax junius, dragonfly	Rana catesbeiana, bullfrog	Growth, Survival
Werner & Anholt 1996 Ecology 77:157-169	Aquatic	Anax junius, dragonfly	Rana clamitans, green frog	Growth, Survival
Werner & Peacor 2006 Ecology 87(2): 347-361	Aquatic	Anax junius, dragonfly	Rana clamitans, green frog	Activity, Growth
Werner 1991 Ecology 72(5): 1709-1720	Aquatic	Anax junius, dragonfly	Rana catesbeiana, bullfrog	Activity, Growth
Werner 1991 Ecology 72(5): 1709-1720	Aquatic	Anax junius, dragonfly	Rana clamitans, green frog	Activity, Growth
Wilder & Rypstra 2004 Env Ent 33(4): 1032-1036	Terrestrial	Tenodera aridifolia sinensis, Chinese praying mantis	Pardosa milvina, wolf spider	Activity, Growth, Survival
Wolff & Davis-Born 1997 Oikos 79: 543-548	Terrestrial	Mustela vison, mink	Microtus canicaudus, grey-tailed vole	Activity, Fecundity
Yamada et al 1998 JEMBE 220: 213-226	Aquatic	Cancer productus, red rock crab	Littorina sitkana, snail	Growth

Reference	System	Predator species (or closest approximation)	Prey species (or closest approximation)	Response variables measured
Ylonen & Ronkainen 1994 Evol Ecol 8: 658-666	Terrestrial	Mustela erminea, stoat	Clethrionomys glareolus, bank vole	Fecundity, Growth
Ylonen 1989 Oikos 55(1): 138-140	Terrestrial	Mustela nivalis nivalis, least weasel	Clethrionomys glareolus, bank vole	Fecundity
